# PROM-based monitoring and alerts reduce post-surgery healthcare utilization of patients undergoing joint replacement: A secondary analysis of the PROMoting Quality RCT

**DOI:** 10.1007/s10198-025-01810-6

**Published:** 2025-08-07

**Authors:** David Ehlig, Lukas Schöner, Alexander Geissler, Laura Wittich, Reinhard Busse, Justus Vogel

**Affiliations:** 1 Chair of Health Economics, Policy and Management, School of Medicine, University of St. Gallen, 9000 St.Gallen, Switzerland; 2 Department of Health Care Management, Technical University of Berlin, Berlin, Germany

**Keywords:** Healthcare utilization, Patient-reported outcome measures, PROM, Value-based healthcare, Hip replacement, Knee replacement, Cost analysis, Healthcare, Resources

## Abstract

**Objectives:**

Healthcare systems increasingly face shortages of medical professionals, and simultaneously experience a rise in demand for healthcare services. In this study, we investigated whether a digital PROM-based monitoring and alert system for hip and knee replacement patients post-surgery can support in decreasing healthcare expenditures and utilization.

**Methods:**

We used data from the randomized controlled trial *PROMoting Quality*, focusing on 546 hip and 492 knee replacement patients from nine German hospitals between October 2019 and December 2020 with available claims data. Patients were equally randomized into two groups: one receiving a PROM-based intervention at 1, 3, and 6 months post-surgery, the other receiving standard care. We compared 1-year post-surgery healthcare utilization using mixed-effects regression models. We further extrapolated the intervention effects to the German healthcare system.

**Findings:**

Results showed post-surgery health expenditure reductions of 7.9% (-318.08, *p* = 0.015) for hip and 7.3% (-386.72, *p* = 0.053) for knee replacements. Significant decreases were observed in outpatient care contacts (-1.51, *p* = 0.005), physiotherapy sessions (-1.65, *p* = 0.037), and number of prescriptions (-2.14, *p* = 0.042) for hip replacements. For knee replacement patients, significant determinants of the cost differences were fewer prescriptions (-3.40, *p* = 0.013) and medical aids (-0.81, *p* = 0.041).

**Conclusion:**

Our findings suggest that digital health interventions can reduce utilization and save scarce healthcare resources. It can be hypothesized that the “being taken care of” effect reduced the need for reassurance of the recovery progress, leading to fewer GP visits and decreased utilization of other healthcare services.

**Supplementary Information:**

The online version contains supplementary material available at 10.1007/s10198-025-01810-6.

## Introduction

Healthcare systems increasingly face shortages of healthcare workers such as physicians and nurses [1, 2], and are under pressure due to ever-increasing healthcare costs [3]. This challenge will not only prevail but intensify in the upcoming years due to demographic changes [2]. For instance, in Germany, the old-age dependency ratio (number of individuals aged 65 or older per 100 people of working age) is predicted to increase from 33 in 2020 to 42 in 2035 [4]. This trend implies a growing demand for healthcare services as an older, more comorbid society will utilize the healthcare system more frequently [5]. Simultaneously, it is likely that this development has a negative effect on the supply of the healthcare workforce, with 48% of medical doctors and 24% of nurses in Germany aged 55 years and older, and projected to retire within the next decade [1]. Without significant increases in productivity, i.e., achieving a higher output over cost ratio, access to and quality of care are likely to suffer.

A potential lever is to use digital technologies to complement and support medical services, thereby unburdening medical staff and increasing productivity [6]. As healthcare systems shift towards a more patient-centered approach, digital technologies are increasingly combined with patient-reported outcome measures (PROMs) to remotely monitor patients post-surgery [7] and to manage chronic diseases [8]. These technologies promise improved outcomes and reduced strain on healthcare workers by decreasing physician visits and invasive treatments. In oncologic settings, such monitoring has proven to decrease mortality, emergency room visits [9] and follow-up physician appointments [10–12]. Orthopedics, particularly hip and knee replacements, represents another promising field for employing digital PROM-based remote monitoring tools. These procedures are frequently performed, projected to increase [13, 14], and offer potential for both outcome improvements [15] and reductions in aftercare utilization and cost decreases [16]. However, research on the impact of PROM-based remote monitoring tools on post-surgery cost and healthcare utilization for hip and knee replacements is limited. While Joseph et al. [16] estimated cost savings from reduced utilization through virtual clinics for joint replacement monitoring, their study relied on simulations rather than actual cost data, and focused on a single center.

This paper, a secondary analysis of the large-scale multi-center *PROMoting Quality* randomized controlled trial (RCT) [17], addresses these research gaps. In the intervention group, patients undergoing hip or knee replacement were monitored via a digital PROM tool, with study nurses at hospitals being alerted in case of adverse recovery pathways within the first year post-surgery. Our analysis aims to assess whether the use of this digital PROM-based monitoring and alert system led to a reduction in healthcare service utilization, specifically in physician contacts and expenditures. We used claims data from 24 German statutory health insurances to isolate the expenditures and appointments of 1,038 enrolled hip and knee replacement patients from hospital discharge until 1-year post-surgery. We conducted comparative analyses and applied a mixed-effect model. Additionally, we extrapolated the intervention’s effects on expenditures and utilization to the entire German healthcare system under different scenarios. Understanding where and how the digital PROM-based monitoring and alert system can enhance efficiency in the healthcare system can help free up resources and save costs that can be redistributed to other areas.

## Methods

### Study design, participants and procedures

Data were drawn from the “PROMoting Quality” trial, a 1-year prospective multicentre two-armed parallel RCT conducted in nine German hospitals. Study participants were adult patients (≥ 18 years) undergoing elective primary hip or knee replacement surgery between October 1 st 2019, and December 31 st 2020. The trial received ethical approval from the Charité Universitätsmedizin Berlin ethics committee and was registered with the German Register for Clinical Studies. All participants provided written informed consent, which could be revoked at any time prior to the end of data collection [17]. Reporting in this article adheres to the Consolidated Standards of Reporting Trials (CONSORT) guidelines [18]. The study protocol is published elsewhere [17].

### Randomisation and masking

Patients were randomized 1:1 to intervention or control. The intervention group received standard care plus PROM-based monitoring and alert intervention; the control group received standard care only. Randomization occurred at discharge via Heartbeat ONE, i.e. the software for primary data collection, with allocation sequence concealment to reduce bias. Study personnel and patients were blinded to group allocation until assignment. Blinding of study nurses was not possible after 1-month follow-up due to intervention requirements, but participants remained unaware of their group throughout the study.

### Procedures

Patients in both groups completed disease-specific and generic PROMs that were digitally collected via the Heartbeat ONE software at hospital admission, discharge, and 12 months post-surgery. Additionally, the intervention group completed digital PROM questionnaires at 1-, 3-, and 6-month intervals post-surgery. In case of non-response to a PROM survey, study nurses sent out reminders via email or telephone to the patients. If a patient’s health significantly declined relative to the prior measurement point or exceeded an absolute PROM threshold, an automated alert notified study nurses, who then contacted the patient to consult on their current health status and the problems associated with the triggered alert. If deemed necessary, the study nurses then referred them to their aftercare physician or specialist. The PROM thresholds that triggered alerts were defined in a consortium of 13 orthopedic physicians using the Delphi technique. For a detailed description of the absolute and relative thresholds see Table 5 in the Appendix.

### Data and outcomes

During the trial, a comprehensive dataset (see also Schöner et al. [19]) was collected, including various disease-specific and generic PROMs, clinical indicators, and detailed patient-level expenditure data from participating statutory health insurances. The set of PROMs included the generic EuroQol five levels five dimensions (EQ-5D-5L) and visual analogue scale (EQ-VAS) for health-related quality of life [20], and the disease-specific Hip Disability and Osteoarthritis Outcome Score Physical Function Short-form (HOOS-PS) [21] and Knee Injury and Osteoarthritis Outcome Score Physical Function Short-form (KOOS-PS) [22] to capture joint-specific functionality. Furthermore, patients’ mental health dimensions were assessed using Patient-Reported Outcomes Measurement Information System (PROMIS) Depression Shortform (PROMIS-D-SF) and Fatigue Shortform (PROMIS-F-SF) [23]. Finally, also an analogue likert pain-scale was included to assess pain in the hip, knee and lower back. The intervention (i.e. alerts) was triggered by exceeding thresholds in EQ-5D-5L, HOOS-PS or KOOS-PS, and PROMIS-D-SF and PROMIS-F-SF (see Tables 5 & 6 in the Appendix).

For the purposes of the present study, the expenditure data were of primary relevance. In this respect, the analysis focused exclusively on the subgroup of patients who consented to the use of their statutory health insurance claims data. Patient-level expenditure data were available in high level of detail for the period spanning from 1-year pre-surgery to 1-year post-surgery. Treatment expenditures and health service utilization data came from the claims data of 24 participating statutory health insurances, i.e. BARMER and 23 of the BKK Dachverband (i.e. the federal association of company health insurances), which collectively cover approximately 18 million people in Germany, representing around 23% of the population.

We examined potential effects of the intervention on the utilization and provision of outpatient medical care (including prescription of drugs, and remedies such as physical therapy), as well as inpatient and outpatient hospital treatments. Data were categorized into six main groups for analysis: outpatient care, outpatient hospital care, inpatient care, prescriptions, remedies, and medical aids.

Outpatient care comprises all services and associated expenditures incurred outside of hospital setting, such as appointments at general practitioners (GPs) or specialists. Outpatient hospital care refers to treatments received in a hospital without an overnight stay. Inpatient care encompasses all services requiring hospitalization, including surgeries, subsequent care, and discharge management. Prescriptions cover services requiring a written direction from a physician, such as medications. Remedies are therapeutic services prescribed by a physician, including e.g., physical therapy or nutritional therapy. Medical aids included e.g., hearing aids, prostheses, wheelchairs, orthopedic supports, and other assistive devices.

### Statistical analysis

A comparative analysis tested for differences between study arms in pre- and post-surgery expenditures and health service utilization. Shapiro–Wilk tests determined the data distribution. For normal distributions, two-sample t-tests were applied, and for non-normal distributions, non-parametric Wilcoxon rank-sum tests, with a two-sided 5% significance level. To mitigate outlier effects, the most extreme 5% of values were winsorized. Post-surgery expenditures were adjusted for pre-surgery spending differences using linear regression. These adjusted 1-year post-surgery expenditures were used in the main analyses, while unadjusted expenditures were used for exploratory analyses in specific cost categories.

Further, a mixed effects modelling approach was employed to assess the intervention’s effect on total adjusted post-surgery expenditures and utilization, as well as on the individual components of outpatient care, outpatient hospital care, inpatient care, prescriptions, remedies, and medical aids. In the regression models, we controlled for confounders, including age, sex, post-surgery mobilization (fast track < 6 h after surgery or conventional care), and BMI, which were treated as fixed effects. Additionally, the treating hospital was included as a random intercept in the model to account for potential clustering effects at the hospital level.

An exploratory deep dive focused on hip replacement patients in key expenditure categories that showed significant differences. Main expenditure categories were divided into sub-categories to identify drivers of cost differences between intervention and control groups. Data preparation and statistical analyses were conducted using STATA version 13.1.

Lastly, the findings were extrapolated to estimate the potential annual effect on the German healthcare system. Average cost of the intervention per patient were retrieved from estimates in Schöner et al. [19], which include personnel costs, software implementation, and user license fees. The extrapolation was based on assumptions in three scenarios:

a *Complete scenario:* Assumes that all hip and knee replacement patients in Germany in 2023 received the intervention. According to the Federal Statistical Office of Germany [24], 273,737 hip and 229,551 knee replacements were performed in 2023. This scenario further assumed a 100% participation rate and a 100% follow-up completion rate.
b * Study scenario:* In alignment with the results from the *PROMoting Quality* trial, this scenario assumed an 80% participation rate and an 85% follow-up completion rate.
c * Real-world scenario*: Based on the NHS’s experience with regularly collecting PROMs for hip and knee replacement patients since 2012, this scenario assumed 70% participation rate for hip and 68% for knee replacement patients, with follow-up completion rates of 58% and 53%, respectively [25].

## Results

### Descriptive statistics

Between October 2019 and December 2020, 7,827 patients were recruited from nine hospitals across Germany (see Figure 5 in the Appendix). After exclusions, the remaining 6,807 patients were randomly assigned to either the intervention or control group. The present cost analysis utilized the data of 1,038 patients insured by one of the participating statutory health insurances who consented to the use of their claims data, comprising 546 hip replacement patients (Intervention: 284, Control: 262) and 492 knee replacement patients (Intervention: 238, Control: 254).

Descriptive statistics are summarized in Table 1. The mean age on the day of the surgery was 66.3 years for patients undergoing hip replacement and 65.7 years for patients undergoging knee replacement. Both groups had a higher proportion of female patients (65.4% for hip, 61.8% for knee). Obesity was present in 32.8% of patients undergoing hip and in 53.1% of patients undergoing knee replacement. Comorbidities were absent in 38.4% of hip patients, compared to 29.0% of knee patients. No significant differences were found between the intervention and control groups for any baseline characteristics.

**Table 1:** Descriptive statistics

	Hip replacement			Knee replacement		
	Intervention	Control	Total	Intervention	Control	Total
	(*N* = 284)	(*N* = 262)	(*N* = 546)	(*N* = 238)	(*N* = 254)	(*N* = 492)
Age						
Mean (SD)	65.7 (10.6)	66.9 (10.2)	66.3 (10.4)	66.0 (8.8)	65.5 (9.8)	65.7 (9.3)
Sex (%)						
Female	186 (65.5)	171 (65.3)	357 (65.4)	149 (62.6)	155 (61.0)	304 (61.8)
Male	98 (34.5)	91 (34.7)	189 (34.6)	89 (37.4)	99 (39.0)	188 (38.2)
BMI (%)						
Underweight	2 (0.7)	2 (0.8)	4 (0.7)	0 (0.0)	0 (0.0)	0 (0.0)
Normal	84 (29.6)	82 (31.3)	166 (30.4)	33 (13.9)	31 (12.2)	64 (12.0)
Overweight	111 (39.1)	86 (32.8)	197 (36.1)	79 (33.2)	88 (34.7)	167 (33.9)
Obese	87 (30.6)	92 (35.1)	179 (32.8)	126 (52.9)	135 (53.2)	261 (53.1)
Mobilization after surgery (%)						
Within 6 h (RR)	135 (47.5)	138 (52.7)	273 (50.0)	124 (52.1)	109 (42.9)	233 (47.4)
Between 6 and 12 h (CC)	81 (28.5)	70 (26.7)	151 (27.7)	72 (30.3)	62 (24.4)	134 (27.2)
Between 12 and 24 h (CC)	61 (21.5)	49 (18.7)	110 (20.2)	34 (14.3)	74 (29.1)	108 (22.0)
Between 24 and 48 h (CC)	2 (0.7)	3 (1.2)	5 (0.9)	6 (2.5)	6 (2.4)	12 (2.4)
After 48 h (CC)	5 (1.8)	2 (0.8)	7 (1.3)	2 (0.8)	3 (1.2)	5 (1.0)
Comorbidities (%)						
None	109 (38.4)	94 (35.9)	203 (37.2)	69 (29.0)	61 (24.0)	130 (26.4)
Arthrosis/Arthritis	51 (18.0)	48 (18.3)	99 (18.1)	49 (20.6)	46 (18.1)	95 (19.3)
Cancer (within last 5 years)	11 (3.9)	16 (6.1)	27 (4.9)	16 (6.7)	16 (6.3)	32 (6.5)
Circulation-disturbances	6 (2.1)	10 (3.8)	16 (2.9)	11 (4.6)	10 (3.9)	21 (4.3)
Depression	16 (5.6)	19 (7.3)	35 (6.4)	17 (7.1)	23 (9.1)	40 (8.1)
Diabetes mellitus	25 (8.8)	26 (9.9)	51 (9.3)	25 (10.5)	35 (13.8)	60 (12.2)
Diseases of nervous system	4 (1.4)	4 (1.5)	8 (1.5)	6 (2.5)	3 (1.2)	9 (1.8)
Heart disease	38 (13.4)	32 (12.2)	70 (12.8)	32 (13.4)	39 (15.4)	71 (14.4)
Hypertension	128 (45.1)	136 (51.9)	264 (48.4)	143 (60.1)	157 (61.8)	300 (61.0)
Kidney disease	8 (2.8)	10 (3.8)	18 (3.3)	6 (2.5)	6 (2.4)	12 (2.4)
Liver disease	5 (1.8)	4 (1.5)	9 (1.6)	6 (2.5)	4 (1.6)	10 (2.0)
Lung disease	26 (9.2)	21 (8.0)	47 (8.6)	23 (9.7)	23 (9.1)	46 (9.3)
Rheumatoid Arthritis	11 (3.9)	13 (5.0)	24 (4.4)	14 (5.9)	29 (11.4)	43 (8.7)
Stroke-related disabilities	7 (2.5)	8 (3.1)	15 (2.7)	5 (2.1)	7 (2.8)	12 (2.4)
PROM baseline score means (SD)						
EQ-5D-5L	0.564 (0.263)	0.599 (0.246)	0.581 (0.255)	0.615 (0.235)	0.606 (0.253)	0.610 (0.244)
EQ-VAS	54.0 (18.5)	55.1 (19.2)	54.5 (18.8)	56.8 (18.5)	57.9 (18.5)	57.3 (18.5)
HOOS-/KOOS-PS	50.3 (16.4)	47.7 (15.3)	49.0 (16.0)	42.6 (11.5)	43.4 (10.9)	43.0 (11.2)
PROMIS-F-SF	48.8 (9.9)	48.5 (10.6)	48.6 (10.2)	47.9 (9.8)	48.2 (9.9)	48.0 (9.9)
PROMIS-D-SF	50.2 (8.4)	49.7 (8.2)	49.9 (8.3)	49.4 (8.5)	49.7 (8.6)	49.6 (8.6)
Pain Score	2.9 (1.5)	2.9 (1.3)	2.9 (1.4)	2.8 (1.3)	2.8 (1.3)	2.8 (1.3)

*BMI* Body Mass Index; *RR* Rapid recovery, i.e. mobilization within 6 h post-surgery; *CC* Conventional Care, i.e. mobilization > 6 h post-surgery; *PROM* Patient-Reported Outcome Measures; *SD* Standard Deviation; *EQ-5D-5L* EuroQol five dimensions, five levels; *EQ-VAS* EuroQol virtual analogue scale; *HOOS-PS* Hip Disability and Osteoarthritis Outcome Score Physical Function Short-form; *KOOS-PS* Knee Injury and Osteoarthritis Outcome Score Physical Function Short-form; *PROMIS* Patient-Reported Outcomes Measurement Information System Depression Shortform (PROMIS‐D‐SF) and Fatigue Shortform (PROMIS‐F‐SF)

**Fig. 1:**
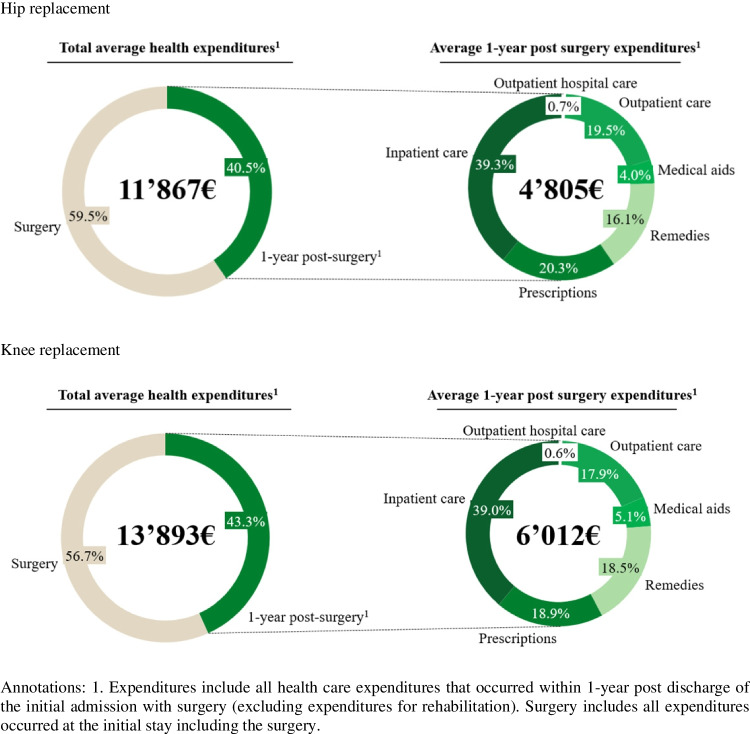
Mean healthcare expenditures per patient for hip and knee replacement from surgery until 1-year post-surgery

Mean expenditures (from the health insurance perspective) from initial admission to 1-year post-surgery are higher for patient undergoing knee than for patients undergoing hip replacement (€13,893 vs. €11,865), with approximately 57 to 60% of the expenditures attributed to initial stay including the surgery itself (see Fig. 1). Similarly, 1-year post-surgery mean expenditures were higher for knee replacement patients (€6,012) compared to hip replacement patients (€4,805).

### Comparative analysis of post-surgery expenditures

#### Hip replacement patients

The intervention group had significantly lower adjusted expenditures of €376.43 (9.4%) compared to the control group (*p* = 0.004, Wilcoxon rank-sum test) over the 1-year post-surgery period. This difference in expenditures was primarily attributed to significant differences in the utilization of outpatient care, inpatient care, prescriptions, remedies, and medical aids (see Table 2).

**Table 2:** Post-surgery healthcare utilization for hip replacement patients

		Intervention (*n* = 284)					Control (*n* = 262)						Comparative statistics^a^
		mean	SD	median	p25	p75	mean	SD	median	p25	p75	Mean Diff.	*p*-value
Outpatient care	Utilization n (%) ^b^	284 (100.00%)					261 (99.62%)						0.298^w^
	Contacts ^c^	21.70	13.17	20	13	27	24.59	15.02	22	14	32	−2.89	0.028^w^
	Raw in € ^d^	852.27	856.52	658.70	403.95	1030.47	1026.72	1075.65	807.92	463.66	1231.61	−174.45	0.003^w^
	Adjusted in € ^e^	816.43	329.89	740.13	568.31	990.41	907.01	360.41	817.18	517.66	1157.52	−90.58	0.002^w^
Outpatient hospital care	Utilization n (%) ^b^	21 (7.39%)					21 (8.02%)						0.786^w^
	Contacts ^c^	0.15	0.63	0	0	0	0.15	0.61	0	0	0	−0.01	0.823^w^
	Raw in € ^d^	32.00	145.72	0.00	0.00	0.00	40.29	217.47	0.00	0.00	0.00	−8.29	0.784^w^
	Adjusted in € ^e^	13.85	6.58	11.60	10.13	15.55	14.67	7.47	12.39	10.07	16.26	−0.82	0.240^w^
Inpatient care	Utilization n (%) ^b^	74 (26.06%)					77 (29.39%)						0.385^w^
	Stays ^c^	0.36	0.71	0	0	1	0.46	0.84	0	0	1	−0.10	0.261^w^
	Raw in € ^d^	1735.18	4404.33	0.00	0.00	437.48	2055.79	4548.14	0.00	0.00	211.16	−320.61	0.352^w^
	Adjusted in € ^e^	1438.52	440.01	1332.26	1107.96	1649.27	1551.83	476.87	1403.10	1184.31	1898.44	−113.31	0.003^w^
Prescriptions	Utilization n (%) ^b^	272 (95.77%)					250 (95.42%)						0.840^w^
	Prescriptions ^c^	14.05	12.69	11	5	19	16.52	14.20	14	6	22	−2.47	0.022^w^
	Raw in € ^d^	745.82	1775.37	280.56	102.11	751.68	1223.32	5054.07	364.97	152.67	842.73	−477.50	0.031^w^
	Adjusted in € ^e^	560.32	586.21	331.51	200.12	645.08	654.50	641.81	404.54	232.68	803.36	−94.18	0.023^w^
Remedies	Utilization n (%) ^b^	239 (84.15%)					227 (86.64%)						0.413^w^
	Prescriptions ^c^	8.36	8.58	6	3	11	10.09	10.01	8	3	14	−1.73	0.041^w^
	Raw in € ^d^	689.03	903.64	463.89	228.23	880.88	867.40	1217.52	563.37	233.00	1076.16	−178.37	0.019^w^
	Adjusted in € ^e^	658.19	294.63	560.95	441.44	775.99	722.09	318.29	622.61	464.34	901.82	−63.90	0.010^w^
Medical aids	Utilization n (%) ^b^	175 (61.62%)					172 (65.65%)						0.329^w^
	Prescriptions ^c^	2.36	3.64	1	0	3	2.66	4.01	1	0	3	−0.30	0.388^w^
	Raw in € ^d^	171.96	408.35	56.31	0.00	173.90	218.69	661.61	61.19	0.00	200.77	−46.73	0.320^w^
	Adjusted in € ^e^	132.82	64.35	112.75	87.57	151.28	146.47	67.95	124.89	95.68	176.33	−13.64	0.005^w^
Total	Raw in € ^d^	**4226.26**	**5575.47**	**2146.55**	**1194.42**	**5140.92**	**5432.22**	**7604.43**	**2614.23**	**1410.99**	**7329.03**	**−1205.96**	**0.019**^w^
	Adjusted in € ^e^	**3620.12**	**1544.80**	**3218.23**	**2521.77**	**4141.57**	**3996.55**	**1656.16**	**3508.19**	**2716.41**	**5028.67**	**−376.43**	**0.004**^w^

^a^Comparative Analysis was conducted at 5% level with two-sided t-tests (p(t)) and, in case of non-normality, with Wilcoxon rank-sum tests (p(w))

^b^If a service in the corresponding category was used at least once in the 1-year post-surgery period

^c^Number of contacts/stays/prescriptions per category in the 1-year post-surgery period. Prescribed remedies can include up to 10 units (e.g., of physiotherapy)

^d^Unadjusted occurred expenditures the 1-year post-surgery period

^e^1-year post-surgery period expenditures adjusted for the baseline differences with winsorized linear regression

In both hip replacement groups, almost all patients received outpatient care services at least once (100% and 99.62% utilization rates). However, significant differences were observed in the mean number of contacts per patient and in the mean and median expenditures. On average, intervention group patients had 2.89 fewer contacts than control group patients (*p* = 0.028, Wilcoxon rank-sum test). Accordingly, adjusted outpatient care expenditures were €90.58 (−10.0%) lower for the intervention group (*p* = 0.002, Wilcoxon rank-sum test). Inpatient care utilization and the number of inpatient stays per patient were balanced between groups. However, intervention group patients incurred €113.13 (−7.3%, *p* = 0.003, Wilcoxon rank-sum test) lower adjusted expenditures than control group patients. On average, intervention group patients had 2.47 fewer prescriptions than control group patients (*p* = 0.022, Wilcoxon rank-sum test). Consequently, this led to an average reduction of €94.18 (−14.4%) in adjusted prescription expenditures per patient in the intervention group (*p* = 0.023, Wilcoxon rank-sum test).

Remedies such as physical therapy were prescribed on average by 1.73 instances less (*p* = 0.041, Wilcoxon rank-sum test) in the intervention group, resulting in €63.90 (−8.8%) lower adjusted expenditures (*p* = 0.010, Wilcoxon rank-sum test).

Finally, a significant difference of €13.64 (−9.3%) in adjusted medical aid expenditures was observed per patient in the intervention group compared the control group (*p* = 0.005, Wilcoxon rank-sum test).

#### Knee replacement patients

Patients undergoing knee replacement in the intervention group had adjusted 1-year post-surgery expenditures averaging €4,907.62 (SD €2,186.07), compared to €5,283.13 (SD €2,271.83) in the control group. This difference of €375.50 (−7.1%) was significant (*p* = 0.034, Wilcoxon rank-sum test). The main contributors to this difference were variations in the utilization of outpatient care, inpatient care, prescriptions and medical aids (see Table 3).

**Table 3:** Post-surgery healthcare utilization for knee replacement patients

		Intervention (*n* = 238)					Control (*n* = 254)						Comparative statistic^a^
		mean	SD	median	p25	p75	mean	SD	median	p25	p75	Mean Diff	*p*-value
Outpatient care	Utilization n (%) ^b^	236 (99.16%)					254 (100%)						0.144^w^
	Contacts ^c^	26.86	16.05	23	15	36	27.57	17.43	24	15	35	−0.71	0.677^w^
	Raw in € ^d^	1027.76	854.72	743.31	455.27	1303.56	1121.89	1263.25	860.37	502.91	1342.26	−94.12	0.329^w^
	Adjusted in € ^e^	965.15	411.98	831.82	664.06	1190.77	1034.26	418.81	949.94	711.21	1251.77	−69.11	0.028^w^
Outpatient hospital care	Utilization n (%) ^b^	22 (9.24%)					36 (14.17%)						0.091^w^
	Contacts ^c^	0.12	0.41	0	0	0	0.25	0.88	0	0	0	−0.13	0.076^w^
	Raw in € ^d^	26.39	142.94	0.00	0.00	0.00	48.74	168.50	0.00	0.00	0.00	−22.35	0.074^w^
	Adjusted in € ^e^	20.72	15.24	15.29	9.70	26.72	23.01	17.56	15.36	10.29	31.94	−2.29	0.315^w^
Inpatient care	Utilization n (%) ^b^	76 (31.93%)					90 (35.43%)						0.415^w^
	Stays ^c^	0.47	0.78	0	0	1	0.55	0.91	0	0	1	−0.08	0.412^w^
	Raw in € ^d^	1948.25	4438.04	0.00	0.00	253.09	2716.69	6867.13	0.00	0.00	2954.12	−768.44	0.304^w^
	Adjusted in € ^e^	1890.02	701.86	1669.25	1373.50	2221.44	2011.77	707.99	1854.54	1443.69	2423.45	−121.75	0.020^w^
Prescriptions	Utilization n (%) ^b^	233 (97.90%)					251(98.82%)						0.421^w^
	Prescriptions ^c^	18.49	14.95	15	8	24	21.88	16.52	19	10	28	−3.39	0.010^w^
	Raw in € ^d^	997.81	2152.47	374.85	187.74	912.64	1261.99	2830.65	535.74	252.53	1073.47	−264.18	0.017^w^
	Adjusted in € ^e^	785.98	791.37	473.29	313.98	925.29	907.93	845.99	560.35	335.75	1146.37	−121.95	0.038^w^
Remedies	Utilization n (%) ^b^	215 (90.34%)					233 (91.73%)						0.588^w^
	Prescriptions ^c^	11.41	9.85	9	4	16	13.12	12.23	10	4	17	−1.71	0.272^w^
	Raw in € ^d^	1019.19	1005.56	708.38	346.50	1380.18	1195.83	1412.72	769.65	383.00	1449.68	−176.64	0.301^w^
	Adjusted in € ^e^	1016.48	473.84	859.83	676.56	1143.02	1053.11	521.89	858.09	684.04	1176.47	−36.63	0.416^w^
Medical aids	Utilization n (%) ^b^	135 (56.72%)					162 (63.78%)						0.110^w^
	Prescriptions ^c^	2.22	3.61	1	0	3	3.08	5.08	1	0	4	−0.86	0.031^w^
	Raw in € ^d^	278.80	808.19	37.83	0.00	225.58	335.27	881.04	81.00	0.00	323.53	−56.47	0.047^w^
	Adjusted in € ^e^	229.28	147.03	178.97	117.97	279.93	253.05	158.45	199.81	128.32	345.75	−23.77	0.093^w^
Total	Raw in € ^d^	**5298.20**	**6041.66**	**3179.92**	**1615.32**	**6208.12**	**6680.41**	**8645.18**	**3555.63**	**1749.12**	**9136.78**	**−1382.20**	**0.074**^w^
	Adjusted in € ^e^	**4907.62**	**2186.07**	**4221.53**	**3307.58**	**5716.67**	**5283.13**	**2271.83**	**4633.52**	**3517.36**	**6478.86**	**−375.50**	**0.034**^w^

^a^Comparative Analysis was conducted at 5% level with two-sided t-tests (p(t)) and, in case of non-normality, with wilcoxon rank-sum tests (p(w))

^b^If a service in the corresponding category was used at least once in the 1-year post-surgery period

^c^Number of contacts/stays/prescriptions per category in the 1-year post-surgery period. Prescribed remedies can include up to 10 units (e.g., of physiotherapy)

^d^Unadjusted occurred expenditures the 1-year post-surgery period

^e^1-year post-surgery period expenditures adjusted for the baseline differences with winsorized linear regression

While no significant differences were found in the number of outpatient care contacts per patient, the adjusted expenditures associated with these contacts were higher for patients in the control group. The adjusted mean total outpatient care expenditures were €69.11 (−6.7%) lower than in the control group (*p* = 0.028, Wilcoxon rank-sum test).

Similarly, no differences in inpatient care utilization or the number of inpatient stays per patient were noted, yet the adjusted mean expenditures in the intervention group were €121.75 (−6.1%) lower than in the control group (*p* = 0.020, Wilcoxon rank-sum test).

On average, patients in the intervention group received 3.39 fewer prescriptions than patients in the control group (*p* = 0.010, Wilcoxon rank-sum test). This reduction was associated with a significant decrease in adjusted prescription expenditures of €121.95 (−13.4%) in the intervention group (*p* = 0.038, Wilcoxon rank-sum test). Finally, a significant deviation was observed in the number of prescribed medical aids. The intervention group averaged 0.86 fewer medical aid prescriptions per patient than in the the control group (*p* = 0.031, Wilcoxon rank-sum test).

### Regression analysis – mixed effect model

#### Hip replacement patients

**Fig. 2:**
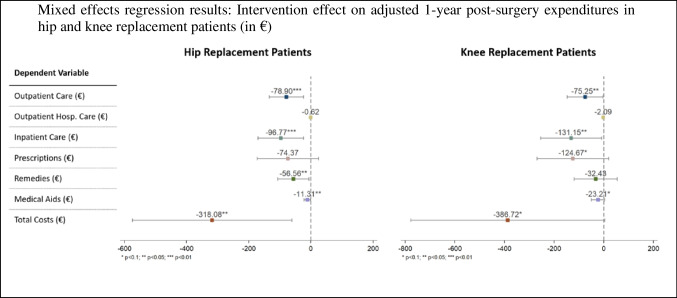
Displays the mixed effect model point estimates of the intervention effect on the adjusted 1-year post-surgery expenditures per hip and knee replacement patient. The lines represent their corresponding 95% confidence intervals. Point estimates above zero would indicate higher expenditures in the intervention group compared to the control group; point estimates below zero indicate lower expenditures in the intervention group compared to the control group. A sensitivity analysis considering the unadjusted expenditures is shown in Fig. 7 in the Appendix, Statistical significance markers: * *p* < 0.1; ** *p* < 0.05; *** *p* < 0.01

**Fig. 3:**
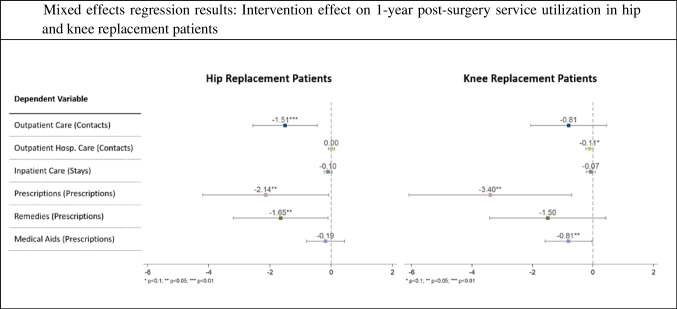
Displays the mixed effect model point estimates of the intervention effect on the 1-year post-surgery per hip and knee replacement patient. The lines represent their corresponding 95% confidence intervals. Point estimates above zero would indicate higher utilization in the intervention group compared to the control group; point estimates below zero indicate lower utilization in the intervention group compared to the control group, Statistical significance markers: * *p* < 0.1; ** *p* < 0.05; *** *p* < 0.01

The left panel of Fig. 2 depicts the point estimates of the intervention effect on the adjusted 1-year post-surgery expenditures per hip replacement patient. The expenditures were significantly lower in the intervention group than in the control group (−318.08, *p* = 0.015, CI −574.82 to −61.33). This reduction in total expenditures was primarily driven by significant differences in outpatient care (−78.90, *p* = 0.005, CI −134.46 to −23.32), inpatient care (−96.77, *p* = 0.010, CI −170.33 to −23.20), remedies (−56.56, *p* = 0.027, CI −106.70 to −6.43), and medical aids (−11.31, *p* = 0.037, CI −21.95 to −0.68). Although a tendency towards savings in favor of the intervention group was observed in prescriptions, this difference was not significant, contrary to the comparative analysis. However, as shown in the left panel of Fig. 3, there are significantly fewer post-surgery prescriptions per patient in the intervention group (−2.14, *p* = 0.042, CI −4.20 to −0.08). Additionally, the intervention group had fewer contacts per patient for outpatient care services (−1.51, *p* = 0.005, CI −2.56 to −0.46) and remedies (−1.65, *p* = 0.037, CI −3.2 to −0.10).

#### Knee replacement patients

The right panel of Fig. 2 shows the point estimates of the intervention effect on the adjusted 1-year post-surgery expenditures per knee replacement patient. The intervention group had lower adjusted total expenditures compared to the control group, though this difference reached statistical significance only at a level of α = 0.1 (−386.72, *p* = 0.053, CI −778.01 to 4.56). This reduction in expenditures was primarily driven by significant differences in adjusted outpatient care expenditures (−75.25, *p* = 0.042, CI −147.68 to −2.81) and in adjusted inpatient care expenditures (−131.15, *p* = 0.037, CI −254.40 to −7.91). Additionally, weakly significant differences were observed in adjusted prescription expenditures (−124.67, *p* = 0.091, CI −269.43 to 20.08) and medical aid expenditures (−23.21, *p* = 0.089, CI −49.99 to 3.56).

While the adjusted expenditure differences in prescriptions and medical aids were only weakly significant, significant differences were found in the number of prescriptions per patient (−3.40, *p* = 0.013, CI −6.09 to −0.71) and the number of prescribed medical aids (−0.81, *p* = 0.041, CI −1.59 to −0.03) (see Fig. 3). Additionally, a weakly significant difference was noted in outpatient hospital care visits (−0.11, *p* = 0.062, CI −0.24 to 0.01).

### Selected expenditure categories within hip replacement patients

#### Outpatient care

One of the main drivers for the significant differences in post-surgery expenditures was the variation in outpatient care contacts. Outpatient care services were categorized into 15 distinct specialist areas (see Table 7 in the Appendix), with the four most relevant categories being GP, internists (INT), orthopedists (ORT), and radiologists (RAD), collectively accounting for approximately 69.7% of all outpatient care expenditures.

**Fig. 4:**
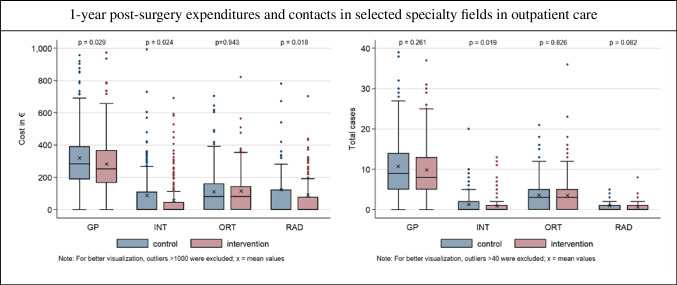
Shows the boxplots of the 1-year post-surgery expenditures and contacts in selected specialty fields in the outpatient sector in hip replacement patients. *p*-values correspond to the results of the Wilcoxon Rank-sum test, Abbreviations: GP – General Practitioner; INT – Internist; ORT – Orthopedist; RAD – Radiologist

**Fig. 5:**
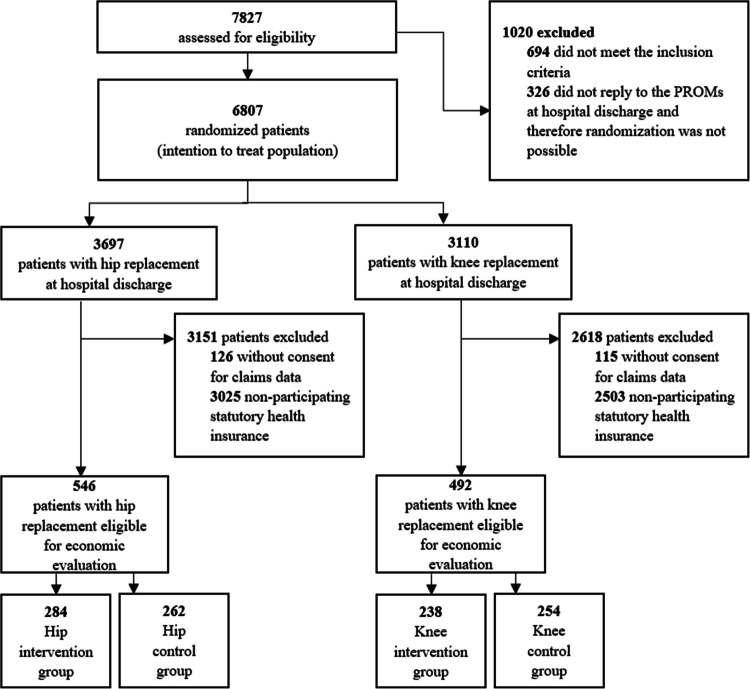
Trial flow chart

The majority of outpatient care expenditures within the 1-year post-surgery period were incurred in the GP category (see Fig. 4). Patients in the intervention group averaged GP expenditures of €283.76 (SD €194.37), while control group patients had significantly higher expenditures of €319.37 (SD €217.62) (*p* = 0.029, Wilcoxon rank-sum test). Similarly, in the INT, the control group had higher expenditures with €87.95 (SD €239.11) compared to the intervention group with €53.90 (SD €114.18) (*p* = 0.024, Wilcoxon rank-sum test). Significant expenditure differences were also observed in RAD, with the control group incurring 122.96€ (SD 745.29) compared to 89.85€ (SD 478.05) in the intervention group (*p* = 0.018, Wilcoxon rank-sum test). For ORT, no significant differences were found.

#### Remedies

**Fig. 6:**
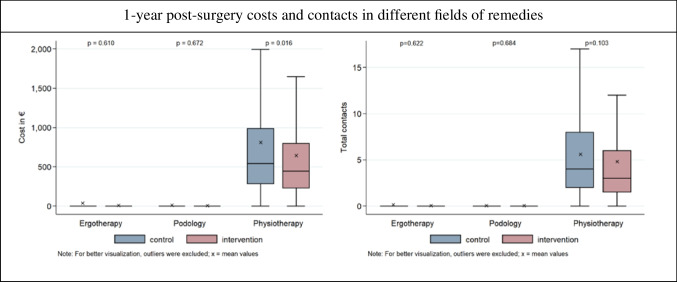
Shows the boxplots of the 1-year post-surgery expenditures and contacts in different fields of remedies in hip replacement patients. P-values correspond to the results of the Wilcoxon Rank-sum test. Other remedies categories such as speech therapy and nutritional therapy were omitted due to absence of observations. In the context of the Covid pandemic, there was another special category of remedies that related to special hygiene measures but is not relevant here and has therefore also been omitted from the visualisation

Another significant driver of post-surgery differences in expenditures between the groups were differences in prescribed remedies. Medical remedies are typically divided into five different forms of therapy: ergotherapy, podology, physiotherapy, speech therapy, and nutritional therapy. Among these, the most substantial differences were observed in physiotherapy (see Figure 6 in the Appendix). The intervention group incurred significantly lower mean expenditures of €645.10 (SD €718.46) compared to the control group with mean expenditures of €803.84 (SD €1,056.37) per patient (*p* = 0.016, Wilcoxon rank-sum test). The remaining therapy forms within remedies showed very low utilization rates and no significant differences between groups.

### German-wide extrapolation

Based on the number of hip and knee replacements performed in Germany in 2023, an extrapolation of our findings suggests potential annual expenditure savings (assuming no cost for the intervention) ranging from €35.2 million to €87.1 million for hip replacements and from €32.2 million to €52.0 million for knee replacement patients, depending on the scenario considered (see Table 4). Intervention costs (including personnel, implementation costs and software licensing fees) within the study are approximated to 123.78€ per patient (see Schöner et al. [19]), which approximately reduces the savings by one third. The lower bound represents a real-world scenario, while the upper bound assumes the complete scenario, where average effects are extrapolated across all hip and knee replacements. Additionally, the scenarios reveal that outpatient care contacts for hip replacement patients could be reduced from 167,176 (real-world scenario) up to 413,343 contacts (complete scenario). Additionally, prescriptions could be reduced from 236,925 (real-world scenario) up to 585,797 (complete scenario). For knee replacement patients, there is a potential reduction of 282,745 (real-world scenario) to 780,473 (complete scenario) in annual drug prescriptions, and 15,800 (reald-world scenario) to 43,615 (complete scenario) fewer medical aid prescriptions.

**Table 4:** Extrapolation scenarios—efficiency gains per cost category for hip and knee replacement patients

		Sign.	Study setting		Scenarios		
			per patient	Intervention group patients	(a) complete	(b) study	(c) real-world
**Hip replacement**							
Patients (n)			1	284	273,737	186,141	110,713
Expenditures savings (study setting in €, scenarios in k€)	Outpatient care	***	78.90	22,408	21,598	14,687	8,735
	Outpatient hosp. care		0.62	176	170	115	69
	Inpatient care	***	96.77	27,483	26,490	18,013	10,714
	Prescriptions		74.37	21,121	20,358	13,843	8,234
	Remedies	**	56.56	16,063	15,483	10,528	6,262
	Medical aids	**	11.31	3,212	3,096	2,105	1,252
Total expenditures savings (study setting in €, scenarios in k€)		**	318.08	90,335	87,070	59,208	35,215
Average intervention costs^a^ (study setting in €, scenarios in k€)			123.78	35,154	33,883	23,041	13,704
Total savings (incl. intervention costs) (study setting in €, scenarios in k€)			194.30	55,181	53,187	36,167	21,511
Reduction in visits (n)	Outpatient care	***	1.51	429	413,343	281,073	167,176
Reduction in visits (n)	Outpatient hosp. care		0.00	0.00	274	186	111
Reduction in stays (n)	Inpatient care		0.10	28	27,374	18,614	11,071
Reduction prescriptions (n)	Prescriptions	***	2.14	608	585,797	398,342	236,925
Reduction in visits (n)	Remedies	**	1.65	469	451,666	307,133	182,676
Reduction prescriptions (n)	Medical aids		0.19	54	52,010	35,367	21,035
**Knee replacement**							
Patients (n)			1	236	229,551	156,095	83,160
Expenditures savings (study setting in €, scenarios in k€)	Outpatient care	**	75.90	17,912	17,423	11,848	6,312
	Outpatient hosp. care		2.09	493	480	326	174
	Inpatient care	**	131.15	30,951	30,106	20,472	10,906
	Prescriptions	*	124.67	29,422	28,618	19,460	10,368
	Remedies		32.43	7,653	7,444	5,062	2,697
	Medical aids	*	23.21	5,478	5,328	3,623	1,930
Total expenditures savings (study setting in €, scenarios in k€)		*	386.72	91,266	88,772	60,365	32,160
Average intervention costs^a^ (study setting in €, scenarios in k€)			123.78	29,212	28,414	19,321	10,294
Total savings (incl. intervention costs) (study setting in €, scenarios in k€)			262.94	62,054	60,358	41,044	21,866
Reduction in visits (n)	Outpatient care		0.81	191	185,936	126,437	67,360
Reduction in visits (n)	Outpatient hosp. care	*	0.11	26	25,251	17,170	9,148
Reduction in stays (n)	Inpatient care		0.07	17	16,069	10,927	5,821
Reduction prescriptions (n)	Prescriptions	**	3.40	802	780,473	530,722	282,745
Reduction in visits (n)	Remedies		1.50	354	344,327	234,142	124,741
Reduction prescriptions (n)	Medical aids	**	0.19	45	43,615	29,658	15,800

Annotations: For better readability, expenditures in study setting are expressed as €, in the scenarios as k€. Effects and statistical significance levels are taken from the mixed-effects models. ^a^ Intervention costs (incl. personnel, implementation costs and software licensing fees) within the study can be approximated to 123.78€ per patient (see Schöner et al. [19]), Scenarios based on all hip and knee replacementsin Germany in 2023: *(a) complete:* 100% participation rate, 100% follow-up completion rate; *(b) study:* 80% participation rate, 85% follow-up completion rate (PROMoting Quality); *(c) real world:* 70 (hip)/68 (knee)% participation rate, 58 (hip)/53 (knee)% follow-up completion rate (NHS Digital) [25], Statistical significance markers: * *p* < 0.1; ** *p* < 0.05; *** *p* < 0.01

## Discussion

This study evaluated the effect of a remote digital health intervention, in particular the use of a PROM-based monitoring and alert system on healthcare expenditures and healthcare resource utilization for patient undergoing hip and knee replacement until 1-year post-surgery. Results are drawn based on data from the large-scale multi-center RCT, *PROMoting Quality*. While prior studies demonstrated that the intervention had a favorable effect on patients’ health outcomes [15, 26], this study’s findings indicated that the intervention also led to savings in expenditures and utilization reductions for both, hip and knee replacement patients, though the extent and statistical significance varied.

For hip replacement patients, the intervention resulted in significant average total cost savings of €318.08 per patient compared to the control group. These savings were largely attributed to significantly fewer outpatient care appointments (1.51), fewer remedies such as physiotherapy (1.65), and fewer drug prescriptions (2.14). Significant expenditure reductions were observed in outpatient care (€78.90), inpatient care (€96.77), remedies such as physiotherapy (€56.56), and medical aids (€11.31). A closer look into outpatient care services revealed that the majority of differences in expenditures and utilization originated from visits to the GP, the INT, and RAD. Contrary to expectations, no significant differences were oberserved in the utilization and costs of orthopedic services from baseline to 1-year post-surgery. As discussed in Steinbeck et al. [15], these effects could be due to patients feeling more adequately cared for, and only visiting their GP for reassurance. This is consistent with the findings by Joseph et al. [16], who showed that a large proportion of patients who requested face-to-face physician appointments after a virtual clinic meeting at 12-month post-surgery mainly sought reassurance about their recovery.

For knee replacement patients, similar patterns were observed, although statistical power was weaker. For instance, the intervention led to a weakly significant expenditure reduction of €386.72 per patient. This difference can partially be explained by significantly fewer prescriptions (3.40) and fewer medical aids (0.19). Significant cost differences between the intervention and control group persisted for outpatient care (€75.90), inpatient care (€131.15), prescriptions (€124.76), and medical aids (€23.21). Interestingly, for both indications significant differences in inpatient care expenditures were observed but no differences in utilization. This can be explained by few more resource intensive treatments in the control in comparison to the intervention group (e.g., re-operations or readmissions [19]) leading to significant differences in inpatient care expenditures but no visible differences in visits.

For orthopedic treatments, there is limited comparable scientific evidence. While different in its study design, Joseph et al. [16] found that follow-ups for hip and knee replacement patients at 12-months using virtual clinics (PROMs and radiographs) to triage face-to-face physician visits could save up to €85 per patient. Approximately 60% of the 154 patients in the study did not require a face-to-face physician visit after a virtual clinic check, and of the remainder, the majority only needed reassurance that their recovery was on track.

These results align with our study’s results – the greater savings potential observed is likely based on the continuous monitoring in our intervention, providing multiple opportunities to save healthcare resources during the 12-months post-surgery period. Similar reductions in healthcare utilization and costs have also been observed in oncologic indications, such as lung cancer or breast cancer [11, 27]. For instance, Riis et al. [11] found that using PROMs to monitor breast cancer patients’ health status and individualize their treatment protocols halved follow-up visits without compromising quality of care.

In line with these findings, our simulation of the intervention indicates substantial potential for reducing healthcare utilization and expenditures. Extrapolation of the effects on expenditures to the entire German health system, in which case volumes in 2023 were 273,737 for hip and 229,551 for knee replacement [24], yielded potential savings between €35 million and €87 million for hip replacements and between €32 million and €52 million for knee replacements, depending on the response rate scenarios. Considering conservative estimations of implementation and operational costs (based on the study intervention costs) reduce the savings by approximately one-third (€21 to €54 million for hip and €22 to €60 million for knee replacements) – offering a lower bound on the savings potential. Moreover, the already overburdened outpatient sector could be relieved by 167 to 413 thousand fewer appointments annually for hip replacement patients alone, offering a promising approach to free-up increasingly scarce human resources (e.g., GPs) and counteract the projected medical workforce shortages [1].

When reflecting upon the effects of the investigated intervention on outcomes and costs, the potential for cost and resource savings achievable with digital PROM monitoring appears especially promising. Due to the intervention, outcomes for hip replacement patients increased by 2.54 points on a 0 (lowest health status) to 100 (highest health status) scale of the PRO-CM score designed for the primary analyses of the PROMoting Quality trial [19]. While the outcome improvement was significant and is not negligible, the potential for cost and resource savings, especially at the national level and given current and futures challenges as discussed above, might constitute the “true” value of digital PROM monitoring tools and similar interventions.

For a wider role-out the acceptance of medical professionals and patients is paramount. For medical professionals, the intervention needs to be efficiently integrated into clinical processes balancing additional efforts by hospitals [28] (e.g., through study nurses) and response rates from patients [29]. However, automation of PROM collection and health monitoring, and their seamless integration into clinical processes might still pose a challenge in German hospitals due to their low level of digital maturity – especially when it comes to patient interaction platforms [30]. For patients, improving acceptance, and thereby response rates, is key. One effective strategy is to provide patients with personalized feedback [31]. Consequentially, alerts during the post-surgery monitoring should only be triggered if it is likely that the individual patient is encountering a problematic recovery.Various studies showed that more personalized alerts based on patient characteristics better reflect individual recovery pathways [32–34] and thus, could lead to a more accepted and effective monitoring. Furthermore, physicians and their communication with the patient regarding PROMs play a crucial role in improving response rates, as a recent study from the Netherlands showed [31], and should therefore be in the center when implementing the intervention.

The study has several limitations. First, fewer than the 1,038 anticipated patients [17] could be included into the study. Nevertheless, with the final sample size, we were able to show significant effects within this study – ruling out potential type 1 error. Moreover, claims data were retrieved from only approximately 15% of the RCT sample due to statutory health insurance association. As sample characteristics of this RCT sample and the studys’ overall sample with all 6,807 patients show very similar patterns (see Steinbeck et al.[15]), we are confident that the results are representative for all hip and knee replacements in the study sample. However, minor differences between the two samples exist in the distribution of gender and rates of obesity. Table 8 in Appendix shows the patient characteristics of the overall sample. Second, due to the complex payment structure of rehabilitation services in Germany – where rehabilitation expenditures for non-retired individuals are covered by the pension insurance – we could not take these expenditures into account when evaluating the effects of the PROM-based monitoring and alert system. However, rehabilitation after hip or knee replacement usually ends before the intervention starts (first PROM collection point 1-month post-surgery) [35], thus, it can be hypothesized that there are no effect due to the intervention. Third, although prescription expenditure reductions were significant for both groups – we could not analyze prescription data in detail due to data access restrictions. A more detailed analyses could have provided insights into the effect on the use of relevant medications post-joint replacement surgery, such as painkillers. Fourth, the intervention consisted of several steps from (1) patients answering the PROM questionnaires at different follow-up times, (2) automated digital alerts when recovery was not as expected at follow-up times, (3) contact with patients by the study nurse, and (4) referrals to outpatient physicians for check-up by the study nurses (see Kuklinski et al.[17], Steinbeck et al.[15], and Schöner et al. [19] for more information). Unfortunately, it was not possible to disentangle which step had the greatest impact on costs and utilization post-surgery, and we can only assess the overall effects of the full intervention. Lastly, we did not consider changes in intervention costs when extrapolating the effects of the intervention to the entire German population. Intervention costs within the study included licensing, implementation and personnel costs were €123.78 per patient (see Schöner et al.[19]). Estimating in the simulation that intervention costs are equal to the costs in the trial (€123.78 per patient) is a conservative assumption as we expect these costs to reduce considerably due to economies of scale when the intervention were rolled out. A study on the roll-out of PROMs for hip and knee replacement patients in the Netherlands, for instance, showed costs per case between €17.33 and €28.81 depending on mode of PROM collection, indication, and hospital case volume [29]. A more sophistic simulation of the effects of this larger German roll-out on intervention costs – also considering different implementation options such as fully digitalized workflows – should be subject of future investigations.

## Conclusion

In conclusion, digital health interventions with patient-centered care elements, such as PROM based monitoring and alert systems, are promising tools for monitoring and steering hip and knee replacement patients post-surgery, thereby reducing aftercare resource utilization. A larger nationwide roll-out and evaluation using real-world data is needed to test its suitability for implementation in standard care. Findings from this study are now discussed among the main German stakeholders to be implemented into standard care [36]. Furthermore, this study can serve as a blueprint for monitoring other conditions, particulalry chronic care patients, to detect poor recovery trajectories potentially mitigating adverse events, and to navigate patients more efficiently through the health system.

## Electronic supplementary material

Below is the link to the electronic supplementary material.

Supplementary file1 (PDF 1772 KB)

Supplementary file2 (PDF 68 KB)

## Figures and Tables

**Fig. 7:**
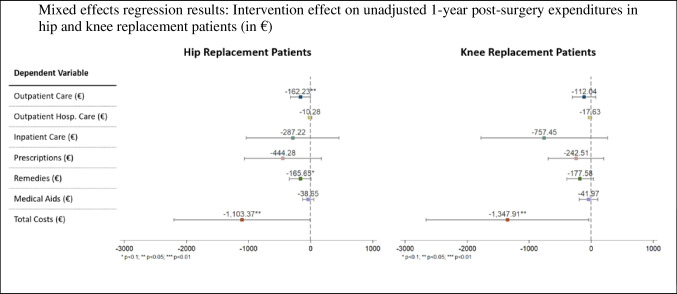
Displays the mixed effect model point estimates of the intervention effect on the 1-year post-surgery expenditures per hip and knee replacement patient. The lines represent their corresponding 95% confidence intervals. Point estimates above zero would indicate higher expenditures in the intervention group compared to the control group; point estimates below zero indicate lower expenditures in the intervention group compared to the control group, Statistical significance markers: * *p*<0.1; ** *p*<0.05; *** *p*<0.01

## Data Availability

Since the data contains sensitive patient information it is legally not allowed to make the data publicly accessible to others due to the German data protection law and the data protection agreements within the trial. In order to enable verifiability of the study results after completion of the project (09/2023), the data will be stored at the research institutions—TU Berlin and aQua Institute—for a period of 10 years after project completion. Data access can only be granted in exceptional cases. Requests for data access should be addressed to the Data Protection Officer at TU Berlin.

## Appendix

See Figs. 5, 6, 7

See Tables 5, 6, 7, 8

**Table 5:** Absolute and relative intervention alert thresholds for each PROM-score

PROM Sets	Month 1^a^	Month 3^a^	Month 6^a^	Relative worsening alert^b^	Relative Threshold
EQ-5D-5L hip	0.37	0.64	0.74	Yes	10% worse than t-1
HOOS-PS	53.00	36.30	27.70	Yes	10pt worse than t-1
EQ-5D-5L knee	0.36	0.51	0.70	Yes	10% worse than t-1
KOOS-PS	51.60	43.60	33.60	Yes	10pt worse than t-1
PROMIS-D-SF	65.80	65.80	65.80	No	NA
PROMIS-F-SF	69.00	69.00	69.00	No	NA

*PROM* Patient-Reported Outcome Measures; *HOOS-PS* Hip Disability and Osteoarthritis Outcome Score Physical Function Short-form; *KOOS-PS* Knee Injury and Osteoarthritis Outcome Score Physical Function Short-form; *PROMIS* Patient-Reported Outcomes Measurement Information System Depression Shortform (PROMIS‐D‐SF) and Fatigue Shortform (PROMIS‐F‐SF)

^a^The absolute alert thresholds were set through a Delphi panel (Kuklinski et al. 2020)

^b^The relative change to a worse score initiated an alert for all PROM-scores with “Yes”. This table is based on the table published in the study protocol of the PROMoting Quality trial (Kuklinski et al. 2020)

**Table 6:** Properties of the PRO measures

PROM	Domain	Range	Directionality^a^
EQ-5D-5L	HRQoL	-0.661–1	Positive
EQ-VAS	HRQoL	0–100	Positive
HOOS-PS	Joint Functionality	0–100	Negative
KOOS-PS	Joint Functionality	0–100	Negative
PROMIS-D-SF	Mental Health	41.0–79.4	Negative
PROMIS-F-SF	Mental Health	33.7–75.8	Negative
Analogue Pain Scales	Pain Symptoms	0–10	Negative

*PROM* Patient-Reported Outcome Measures; *HOOS-PS* Hip Disability and Osteoarthritis Outcome Score Physical Function Short-form; *KOOS-PS* Knee Injury and Osteoarthritis Outcome Score Physical Function Short-form; *PROMIS* Patient-Reported Outcomes Measurement Information System Depression Shortform (PROMIS‐D‐SF) and Fatigue Shortform (PROMIS‐F‐SF); *HRQoL* Healt-Related Quality of Life

^a^A positive directionality indicates that higher scores reflect better outcomes while with a negative directionality a higher score indicates worse symptoms, i.e. bad outcomes

**Table 7:** Outpatient specialist areas

1	General practitioner
2	Ophthalmology
3	Surgery
4	Orthopedics
5	Gynecology
6	ENT (Ear, Nose, Throat)
7	Dermatology
8	Internal medicine
9	Pediatrician
10	Laboratory medicine
11	Neurology
12	Pathology
13	Radiology
14	Urology
15	Psychology

**Table 8:** Descriptive statistics of the entire study sample

	Hip replacement patients		Knee replacement patients	
	Intervention (*N* = 1,854)	Control (*N* = 1,843)	Intervention (*N* = 1,564)	Control (*N* = 1,546)
Age				
Mean (SD)	65.9 (10.6)	65.7 (10.6)	66.1 (9.09)	65.9 (9.36)
Gender (%)				
Female	1,029 (55.5)	1,036 (56.2)	839 (53.6)	830 (53.7)
Male	825 (44.5)	807 (43.8)	725 (46.4)	716 (46.3)
bmi group (%)				
Underweight	10 (0.5)	10 (0.5)	3 (0.2)	3 (0.2)
Normal	576 (31.1)	588 (31.9)	255 (16.3)	222 (14.4)
Overweight	722 (38.9)	699 (37.9)	566 (36.2)	618 (40.0)
Obese	546 (29.4)	546 (29.6)	740 (47.3)	703 (45.5)
Current smoker (%)				
No	1,586 (85.5)	1,547 (83.9)	1,369 (87.5)	1,336 (86.4)
Yes	268 (14.5)	296 (16.1)	195 (12.5)	210 (13.6)
Education (%)				
No school degree	8 (0.4)	7 (0.4)	13 (0.8)	5 (0.3)
Primary school degree	243 (13.1)	249 (13.5)	263 (16.8)	271 (17.5)
High/middle school degree	1,085 (58.5)	1,033 (56.1)	932 (59.6)	927 (60.0)
University degree	518 (27.9)	554 (30.1)	356 (22.8)	343 (22.2)
Living situation (%)				
Alone	430 (23.2)	414 (22.5)	310 (19.8)	289 (18.7)
Care facility	4 (0.2)	7 (0.4)	10 (0.6)	9 (0.6)
With a partner/family/friends	1,411 (76.1)	1,408 (76.4)	1,239 (79.2)	1,233 (79.8)
Other	9 (0.5)	14 (0.8)	5 (0.3)	15 (1.0)
Job (%)				
Working	583 (31.4)	618 (33.5)	454 (29)	468 (30.3)
Voluntarily not working including retirement	1,065 (57.4)	1,034 (56.1)	915 (58.5)	875 (56.6)
Looking for work	20 (1.1)	15 (0.8)	11 (0.7)	23 (1.5)
Unable to work	186 (10)	176 (9.6)	184 (11.7)	180 (11.6)
Mobilization after surgery (%)				
Within 6 h	854 (46.1)	854 (46.3)	716 (45.8)	709 (45.9)
Within 12 h	560 (30.2)	496 (26.9)	460 (29.4)	425 (27.5)
Within 24 h	403 (21.7)	443 (24.0)	331 (21.2)	356 (23.0)
Within 48 h	26 (1.4)	35 (1.9)	43 (2.7)	46 (3.0)
After 48 h	11 (0.6)	15 (0.8)	14 (0.9)	10 (0.6)
Readmission (%)				
No	1,809 (97.6)	1,789 (97.1)	1,531 (97.9)	1,506 (97.4)
Within 30 days post-surgery	19 (1.0)	28 (1.5)	9 (0.6)	21 (1.4)
30–90 days post-surgery	26 (1.4)	26 (1.4)	24 (1.5)	19 (1.2)
Reoperation within 12 months post-surgery (%)				
No	1,816 (98.0)	1,798 (97.6)	1,530 (97.8)	1,504 (97.3)
Yes	38 (2.0)	45 (2.4)	34 (2.2)	42 (2.7)
PROM baseline score means (SD)^a^				
EQ-5D-5L	0.594 (0.263)	0.603 (0.256)	0.625 (0.257)	0.623 (0.245)
EQ-VAS	56.5 (20.0)	57.1 (19.7)	58.7 (19.4)	57.9 (19.1)
HOOS/KOOS-PS	48.4 (16.4)	47.1 (16.1)	43.1 (13.3)	43.0 (12.0)
PROMIS-fatigue	49.2 (9.91)	49.2 (9.98)	48.3 (10.1)	48.1 (9.54)
PROMIS-depression	49.7 (8.35)	49.8 (8.26)	49.4 (8.39)	49.4 (8.16)

*BMI* Body Mass Index; *PROM* Patient-Reported Outcome Measures; *SD* Standard Deviation; EQ-5D-5L EuroQol five dimensions, five levels; *EQ-VAS* EuroQol virtual analogue scale; *HOOS-PS* Hip Disability and Osteoarthritis Outcome Score Physical Function Short-form; *KOOS-PS* Knee Injury and Osteoarthritis Outcome Score Physical Function Short-form; *PROMIS* Patient-Reported Outcomes Measurement Information System Depression Shortform (PROMIS‐D‐SF) and Fatigue Shortform (PROMIS‐F‐SF)
